# Null allele, allelic dropouts or rare sex detection in clonal organisms: simulations and application to real data sets of pathogenic microbes

**DOI:** 10.1186/1756-3305-7-331

**Published:** 2014-07-15

**Authors:** Modou Séré, Jacques Kaboré, Vincent Jamonneau, Adrien Marie Gaston Belem, Francisco J Ayala, Thierry De Meeûs

**Affiliations:** 1 Centre International de Recherche-Développement sur l’Elevage en zone Subhumide (CIRDES), 01 BP 454 Bobo-Dioulasso 01, Burkina-Faso; 2 Université Polytechnique de Bobo-Dioulasso, 01 BP 1091 Bobo-Dioulasso 01, Burkina-Faso; 3 Interactions hôtes - vecteurs - parasites dans les infections par des trypanosomatidae - (INTERTRYP), UMR IRD/CIRAD 177, TA A-17/G, Campus International de Baillarguet, 34398 Montpellier Cedex 5, France; 4 Department of Ecology and Evolutionary Biology, University of California, Irvine, CA 92697-2525, USA

**Keywords:** Population genetics, Clonal reproduction, Allelic dropouts, Null alleles, Heterozygosity, Genetic diversity, Yeasts, Trypanosomes

## Abstract

**Background:**

Pathogens and their vectors are organisms whose ecology is often only accessible through population genetics tools based on spatio-temporal variability of molecular markers. However, molecular tools may present technical difficulties due to the masking of some alleles (allelic dropouts and/or null alleles), which tends to bias the estimation of heterozygosity and thus the inferences concerning the breeding system of the organism under study. This is especially critical in clonal organisms in which deviation from panmixia, as measured by Wright’s *F*_IS_, can, in principle, be used to infer both the extent of clonality and structure in a given population. In particular, null alleles and allelic dropouts are locus specific and likely produce high variance of Wright’s *F*_IS_ across loci, as rare sex is expected to do. In this paper we propose a tool enabling to discriminate between consequences of these technical problems and those of rare sex.

**Methods:**

We have performed various simulations of clonal and partially clonal populations. We introduce allelic dropouts and null alleles in clonal data sets and compare the results with those that exhibit increasing rates of sexual recombination. We use the narrow relationship that links Wright’s *F*_IS_ to genetic diversity in purely clonal populations as assessment criterion, since this relationship disappears faster with sexual recombination than with amplification problems of certain alleles.

**Results:**

We show that the relevance of our criterion for detecting poorly amplified alleles depends partly on the population structure, the level of homoplasy and/or mutation rate. However, the interpretation of data becomes difficult when the number of poorly amplified alleles is above 50%. The application of this method to reinterpret published data sets of pathogenic clonal microbes (yeast and trypanosomes) confirms its usefulness and allows refining previous estimates concerning important pathogenic agents.

**Conclusion:**

Our criterion of superimposing between the *F*_IS_ expected under clonality and the observed *F*_IS_, is effective when amplification difficulties occur in low to moderate frequencies (20-30%).

## Background

The improvement of DNA amplification techniques during the last few decades has had major consequences in the investigation of the genetics of natural populations, in particular populations of pathogens and their vectors, for which direct observation of individuals is difficult or impossible [1]. The use of variable genetic markers in space and time allows inferring basic ecological parameters, such as reproduction unit size, dispersal, spatial organization (structure) of the populations, and mode of reproduction [1-4]. Knowledge of these parameters can be crucial for understanding the epidemiology of pathogenic agents, for evaluating risks of resistance genes or re-invasion after elimination of pathogens and/or of their vectors [5]. However, although parasitic organisms represent a significant part of described species [6] and despite the recent explosion of molecular studies, population studies of host-parasite systems are still rare [4].

Wright [7] built a set of indices, the so called *F*-statistics, which measure the relative contribution of individuals, subpopulations and total populations to inbreeding. *F*-statistics allow discriminating among the different parameters responsible for inbreeding at different levels, such as breeding system and population subdivision. Three coefficients, corresponding to the three hierarchical levels that are individual, subpopulation and total population, are conventionally defined: *F*_IS_, *F*_ST_ and *F*_IT_. *F*_IS_ estimates the amount of inbreeding in individuals relative to the subpopulation, resulting from the reproductive system. *F*_ST_ estimates the inbreeding of subpopulations relative to the total population; it arises from population subdivision into sub-units of limited size with limited exchange (migration). This index is therefore also used for assessing genetic differentiation between subpopulations. *F*_IT_ estimates the inbreeding of individuals relative to the total population, resulting from the combined effects of the previous two. *F*_IS_ varies from −1 to +1, with 0 corresponding to a random assortment of gametes within subpopulations (local panmixia). Negative values correspond to heterozygote excess as would be expected in clones [8] and positive values indicate homozygote excess as would be expected in selfing organisms. *F*_ST_ varies from 0 to 1; 0 corresponds to absence of subdivision (free dispersal between subpopulations) and 1 to maximum differentiation (each subpopulation is fixed for one or other of the available alleles).

Parasitic organisms represent a major part of biodiversity [5,6]; a large part are clonal or partially so, in particular those affecting humans [1,5]. Clonal organisms are expected to display strong excess of heterozygotes and hence strongly negative *F*_IS_ values across the whole genome [8]. This trend is quickly reversed by low rates of recombination, so that *F*_IS_ quickly reaches its expected panmictic value (*F*_IS_ = 0), except when the rates of recombination are very low (*e.g.* 0.0001- 0.05), in which case, a large variance is observed between loci [8]. This variance has been proposed as a useful criterion for detecting very low rates of recombination [9]. However, technical difficulties arise when heterozygosity is hidden (allelic dropouts and/or null alleles). Hidden alleles generally are locus specific and typically result in high variance of *F*_IS_ across loci [1,9]. In strictly clonal organisms, the presence of hidden alleles may thus yield similar observations as very low levels of sexual recombination [9]. Consequently, the presence of allelic dropouts and/or null alleles in a data set brings ambiguity when seeking to ascertain the reproductive system of a population. Therefore, in case of high variance of *F*_IS_ across loci with negative mean, being able to discriminate between hidden alleles and infrequent recombination is an important goal for the study of clonal populations.

In this paper, we propose a new tool for detecting allelic dropouts and null alleles in population genetics data sets of clonal organisms. We propose a simulation approach to investigate different population structures (island, stepping stone), different types of markers (microsatellites, allozymes or SNPs), different rates of clonal reproduction, different rates of null alleles or allelic dropouts and check how our criterion, based on the relationship between *F*_IS_ and genetic diversity, can help to discriminate between rare sex and hidden alleles. We then apply the criterion to various real data sets regarding parasitic microbes: a yeast (*Candida albicans*) (allozymes) and four species of trypanosomes (microsatellite loci). In light of our results, we propose a useful criterion that will allow detection when variance of *F*_IS_ across loci can come from amplification problems and thus when it can be worthwhile eliminating problematic loci, repeating DNA amplification of homozygous and/or missing profiles and/or redesigning primers.

## Methods

### Ethical statement

All data used in the present work were either generated ex-silico or have already been published in peer reviewed journals where ethical statements have already been provided. There is thus no ethical issue associated with our paper.

### The model

*F*_IS_ is typically expressed in terms of the probability of identity between alleles [10,11]: *Q*_I_ represents the probability of identity within individuals and *Q*_S_ is the probability of allelic identity between individuals of the same subpopulation. These identities are by descent for the Infinite Allele Model (IAM) and by state for the *K* Allele Model (KAM).

(1) $$F_{\mathrm{IS}}=\frac{Q_{I}-Q_{S}}{1-Q_{S}}$$

Under the assumption of clonal reproduction, and if the number of possible alleles (*K*) is big enough, then it was shown that all loci tend to become and stay heterozygous [8], hence *Q*_I_ ~ 0 and equation (1) becomes:

(2) $$F_{\mathrm{IS}}=\frac{-Q_{S}}{1-Q_{S}}$$

Knowing that genetic diversity *H*_*S*_ (which represents the probability of non-identity) is the opposite of *Q*_S_ and *Q*_S_ = 1-*H*_*S*_, we have (in clones):

(3) $$F_{\mathrm{IS}}=-\frac{1-H_{S}}{H_{S}}$$

It can be argued that in the case of substantial homoplasy, the approximation of *H*_*s*_ as 1-*Q*_S_ no longer holds. This is probably true but, as will be seen further, this does not have much effect on our results.

### Simulations

The simulated data were generated using EasyPop v2.01 software [12]. We simulated diploid individuals in non-overlapping generations and distributed them in 100 subpopulations of 50 individuals each. The choice of these numbers was made without fundamental principles. This, however, allowed exploring various kinds of population structure with reasonable effects of drift and migration. We simulated 20 loci with mutation rates ranging from *u* = 10^−9^ to *u* = 10^−3^. These mutation rates were selected as regard to the types of commonly used genetic markers such as SNPs, allozymes and microsatellite markers. The mechanism of mutation follows a KAM, where each of *K* possible alleles (1 to *K*) can mutate into any of the *K*-1 available alleles. Each simulation started with a maximum diversity (all *K* alleles evenly distributed among the 100 × 50 individuals) and ended after 10,000 generations, which was enough to reach an approximate equilibrium state [8]. Homoplasy was controlled by varying *K* from 2, 5 and 99 possible allelic states in order to be consistent with the different markers we used as examples: SNPs, allozymes (for which homoplasy is substantial) and microsatellite markers (weak homoplasy). In fact, microsatellite loci displaying many alleles are (by definition) subjected to weak homoplasy even under a strict stepwise mutation model (SMM). Moreover, most microsatellite loci do not follow a strict SMM, in which case any homoplasy signature totally disappears so long as the number of alleles is more than 2 (see [13,14]). Five major groups of simulations were defined as regard to clonal rate *c*: 100%, 99.99%, 99.9%, 99% and 95%. These clonal rates are indeed known to generate *F*_IS_ values different from those expected under panmixia. In each of these five major groups of simulations, three types of population models were explored: island models [15], stepping stone models in one dimension (linear), and stepping stone models in two dimensions [16]. In stepping stone models, migration occurs between adjacent populations, which globally results in more strongly structured populations compared to the island models, especially for one dimension stepping stones [17]. We then considered different migration rates depending on population models: *m* = 0.01 and *m* = 0.5 for the island model, m = 0.5 for stepping stone in one dimension, and m = 0.05 for stepping stone in two dimensions. Finally, each simulation (corresponding to a particular set of parameters) was repeated 10 times (10 replicates). For each replicate, 10 subpopulations and 20 individuals per subpopulation were sampled and submitted to our manipulation and analyses.

Much more diverse parameter sets could have been explored in terms of population structure. Nevertheless, the few variations in population structure we have explored tended to demonstrate that the criterion we used for discriminating rare sex from hidden alleles will not be critically affected by population structure (see Results). Hence our final recommendations can confidently be generalized to most kinds of clonal populations.

### Allelic dropouts and null alleles

An allelic dropout occurs when the PCR (Polymerase Chain Reaction) defined for a given locus fails to amplify one or both alleles of a diploid individual. In the case where only one allele drops out, only one allele (band or peak) is then revealed and the individual is thus misinterpreted as homozygous at the concerned locus. This is a random event (any of the two alleles is as likely to undergo the phenomenon) that generally occurs when the DNA amount is limiting. This phenomenon is more likely to occur when primers do not perfectly match the flanking sequences, as is often the case when these primers have been designed from closely related species or other populations. Allelic dropouts are thus expected to be locus specific most of the time. Allelic dropout can also cause missing genotypes (if both alleles drop out) [18]. Two different kinds of allelic dropouts where investigated. The first model (Dropout 1) could be called competitive allelic dropout where allelic dropout occurs as a result of competition for the Taq polymerase. In that case the phenomenon does not normally generate missing data. This model corresponds to the classical view [19-21], though it was also allele specific in our case (where it could also be assimilated to partial null alleles). Here, for *K* = 99, alleles 1 to 10 (10%), 1 to 20 (20%), 1 to 30 (30%) or all even numbered alleles (50%) were masked when heterozygous with another allele. Individuals heterozygous for two of these alleles at a given locus were coded homozygous for the first allele. For simulations with *K* < 99, allelic dropouts involved a proportionate number of alleles according to the desired percentage and following the same principle as described for *K* = 99. With that model of allelic dropout (or partial nulls), loci that did not keep those alleles that we defined as dropouts at the end of simulation did not display any dropout. We thus did not need to manipulate the data further to generate the desired variance across loci pattern. For the second method (Dropout 2), dropout was stochastic [18]. Simulated data were transformed so that dropouts occur randomly, even at both alleles of an individual [22]. Because the phenomenon should be locus-specific, and in order to vary the proportion of allelic dropouts, the first 2 (for 10%), 5 (for 25%), and half (50%) of the 20 loci were chosen to display allelic dropouts. First, we sorted the whole data set according to allele values of the concerned locus. Then, regardless of subpopulations, at this single concerned locus, the first 25% individuals remained unchanged; the second 25% were coded as missing data (blanks), the third 25% as homozygous for the first allele and the last 25% as homozygous for the second allele. Then, the data were sorted back according to subpopulation value. We have undertaken this process independently for each concerned locus. Since allele labeling results from a random process, this allele dropout hence can also be assimilated to a random process.

Null alleles are defined as alleles that do not produce amplification by PCR. An individual may be homozygous or heterozygous for different alleles. It can be heterozygous for a null allele with one amplified allele, in which case the individual will be perceived as homozygous for the amplified allele, it can be a null homozygous, in which case it corresponds to missing data (no amplification or blank genotype) or it can be homozygous or heterozygous for amplified alleles. The proportion of nulls was controlled as for the Dropout 1 model, except for null individuals harboring two null alleles at the same locus, which were coded as missing data (blank individuals at the concerned locus). Here again, because not all loci displayed the selected alleles at the end of simulation, null alleles did not affect all loci equally, hence producing a random locus specific phenomenon.

Fixation indices were estimated with Weir and Cockerham’s unbiased estimators [23]. Genetic diversity was estimated by Nei’s unbiased estimator (*H*_*s*_) [24]. We estimated these different statistics using the software Fstat v2.9.4 [25], updated from [26].

*F*_IS_ calculated according to equation (3) was named “expected *F*_IS_” (*F*_IS_exp_). *F*_IS_ derived from *F*_IS_ estimated with Fstat from Easypop outputs (with sexual or clonal reproduction, with or without allelic dropouts or null alleles) and from real data sets, was named “observed *F*_IS_” (*F*_IS_obs_). To assess a match between *F*_IS___exp_ and *F*_IS___obs_ we calculated Δ*F*_IS_ = *F*_IS___exp_-*F*_IS___obs_. We then considered that the two values were superimposed when |Δ*F*_IS_| ≤ 0.05 × |*F*_IS_exp_|. Thus, the proportion of superimposed points and its confidence interval at 95%, computed over the 10 replicates of each simulation, were noted for each simulation to serve as a criterion for distinguishing between consequences of hidden alleles (null alleles or allelic dropouts) and sexual recombination. It can be noticed at this stage that other criteria were explored during preliminary studies. In particular, correlation methods connecting *F*_IS_exp_ and *F*_IS_obs_ were analyzed and presented quite poor efficiencies as compared to the criterion expounded above. When *H*_*S*_ < 0.5, equation (3) generates an expected *F*_IS_ < −1. In pure clones, *H*_*s*_ is not expected to be below 0.5, especially so when the number of alleles *K* becomes substantial, but null alleles, allelic dropouts and the presence of sex (even rare) can generate data with several *H*_*s*_ < 0.5. A first exploration of simulated data (Additional file 1: Figure S1) showed that removing those cases where *H*_*s*_ < 0.5 provided much better discrimination between rare sex and hidden alleles. We thus only considered data (loci and subpopulations) for which *H*_*s*_ ≥ 0.5.

### Real data sets

These data sets were chosen among clonal (or supposedly so) organisms, with available genotypic data and displaying possible hidden alleles and/or signature of rare recombination events. For *C. albicans*[27], 14 allozymes were used, half of which were suspected to display null alleles and eventually removed from the analysis by the authors in order to refine the estimate of *F*_IS_. The data of *T. brucei gambiense*[28] concerned six microsatellite loci amplified from extracts of biological fluids (blood, lymph and cerebrospinal fluid). These data showed an unusually high number of homozygotes compared to strictly clonal populations, and particularly relative to the results obtained for the same sites but with DNA amplified mainly after isolation techniques [29]. These results might reflect either the existence of rare and recent sexual events, or more likely amplification problems [28]. Other data from African trypanosomes, the DNA of which was amplified directly from host blood (no isolation step), were also investigated. *T. evansi* from Sudan, the reproductive system of which remains unclear, though assumed to be clonal [30,31], was suspected to present many allelic dropouts, due to the presence of an abnormally high proportion of homozygous individuals without missing genotypes and substantial variance of *F*_IS_ across loci, together with a Wahlund effect [32]. In *T. congolense*, strong heterozygote deficits were found [33], for which the authors proposed a highly inbred sexual mode of reproduction. Nevertheless, the data displayed many missing data. Finally, *T. vivax* data [34] were assumed by authors to fit with expectations under clonal reproduction despite a large variance of *F*_IS_ from one locus to another. We evaluated the proportion of superimposed *F*_IS_ for each of these data sets. The values obtained were compared with those of simulated populations under different modes of migration and reproduction. *C. albicans*, *T. brucei, T. congolense* and *T. vivax* data were compared with simulations corresponding to an island migration model, which seems to fit better [27,29], while *T. evansi* data were compared with a two-dimension stepping stone model [32]. We also conducted a theoretical estimate of the proportion of null alleles and the number of homozygotes as a function of the observed proportion of blank genotypes. The expected number of homozygous genotypes was then compared to the observed one in the *T. brucei* and *T. congolense* data sets, by an exact binomial test using the software R v2.12.0 [35]. For *T. congolense*, we also built a dendrogram based on Cavalli-Sforza and Edwards chord distance [36] with the software MSA v 4.05 [37] and built a Neighborjoining tree (NJTree) using MEGA v3.1 [38].

For each replicate (for the simulation data), we estimated the average of superimposed points over the 10 subpopulations, we then calculated the 95% confidence interval based on the variance between different replicates. For the real data, we only estimated the average of superimposed points over the different available subsamples and calculated the confidence interval based on the variance between them.

## Results

### Influence of rare sex and migration on the proportion of superimposed *F*_IS_

**Figure 1:**
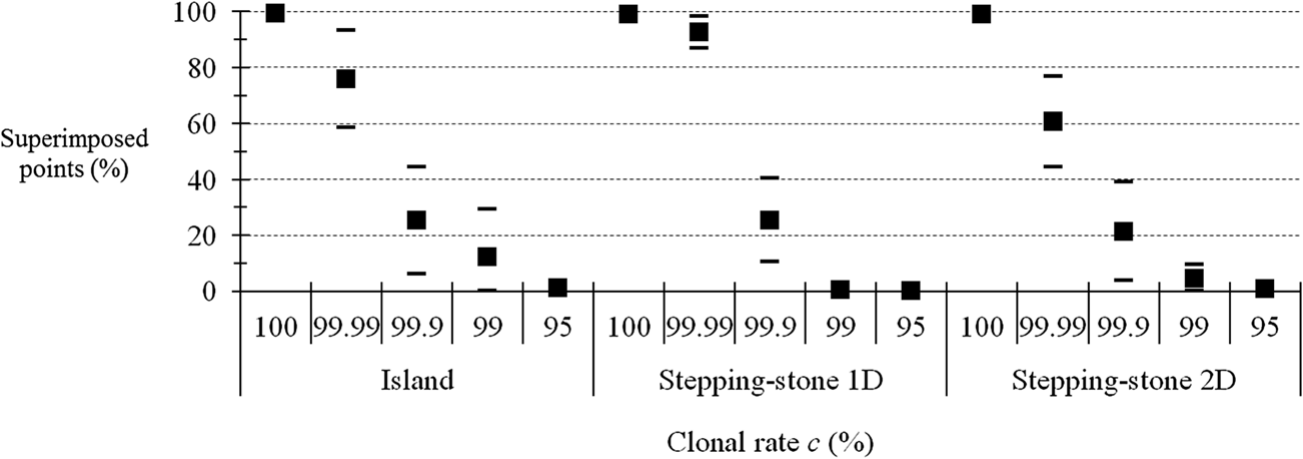
**Proportion of superimposed points (in percent) between expected and observed *F***_**IS **_**for different levels (percent) of clonality (*c *) in different migration models: island model (Island) with *m*** **= 0.01 (migration rate), one-dimension stepping stone model (Stepping-stone 1D) with *m*** **= 0.5, and two-dimension stepping stone model (Stepping-stone 2D) with *m*** **= 0.05.** The maximum number of alleles per locus was *K* = 99 and the mutation rate was *u* = 10^−5^.

The results are shown in Figure 1. We observed that the superposition is almost total for entirely clonal populations (*c* = 100%), regardless of the migration model. We also found that the proportion of superimposed points strongly decreases with rare sex, even with *c* = 99.99% (though to a lesser extent) and becomes as low as 20% with *c* = 99.9%. In all cases, the superimposition becomes practically zero beyond 5% of sex and remains around 10% in the island migration model, and 1% in the stepping stone migration model for 1% of sex. These differences (a priori) between models of migration may be mainly due to the choice of migration rate, rather than being mostly due to the single effect of pattern of migration, as shown below.

### Effects of migration rate and rare sex behavior

**Figure 2:**
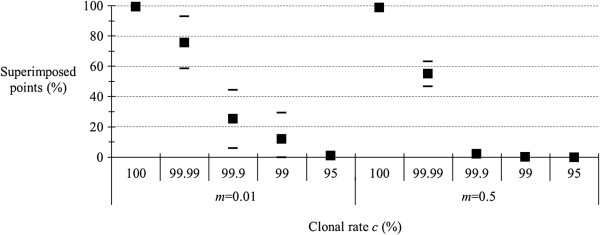
**Proportion of superimposed points (in percent) between expected and observed *F***_**IS **_**for different levels (percent) of clonality (*c*), for different migration rates (*m*) in an island model with *K*** **= 99 and *u*** **= 10**^**−5**^**.**

The results are shown in Figure 2. Obviously, signature of very rare (1/10,000) sex will be less easily seen in strongly subdivided populations.

### Homoplasy

**Figure 3:**
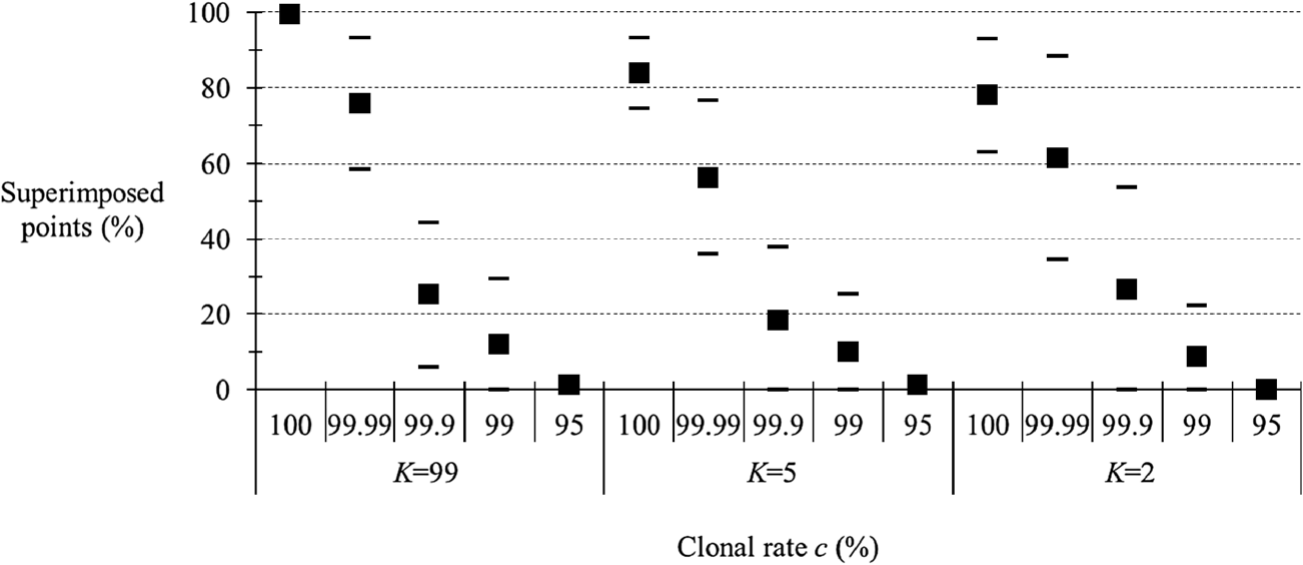
**Proportion of superimposed points (in percent) between expected and observed *F***_**IS **_**for different levels (percent) of clonality (*c*) for different degrees of homoplasy: low (*K*** **= 99), medium (*K*** **= 5) and maximum (*K*** **= 2) in an island model with *u*** **= 10**^**−5 **^**and *m*** **= 0.01.**

The results are presented in Figure 3. We note that when homoplasy is substantial (*K* = 5, *K* = 2), superimposition significantly decreases. However, this effect deserves to be confirmed by adjusting the effect of the mutation rate which is likely to be negatively correlated with homoplasy: markers with low homoplasy have in principle higher mutation rates than markers with high homoplasy.

### Mutation rate and homoplasy

**Figure 4:**
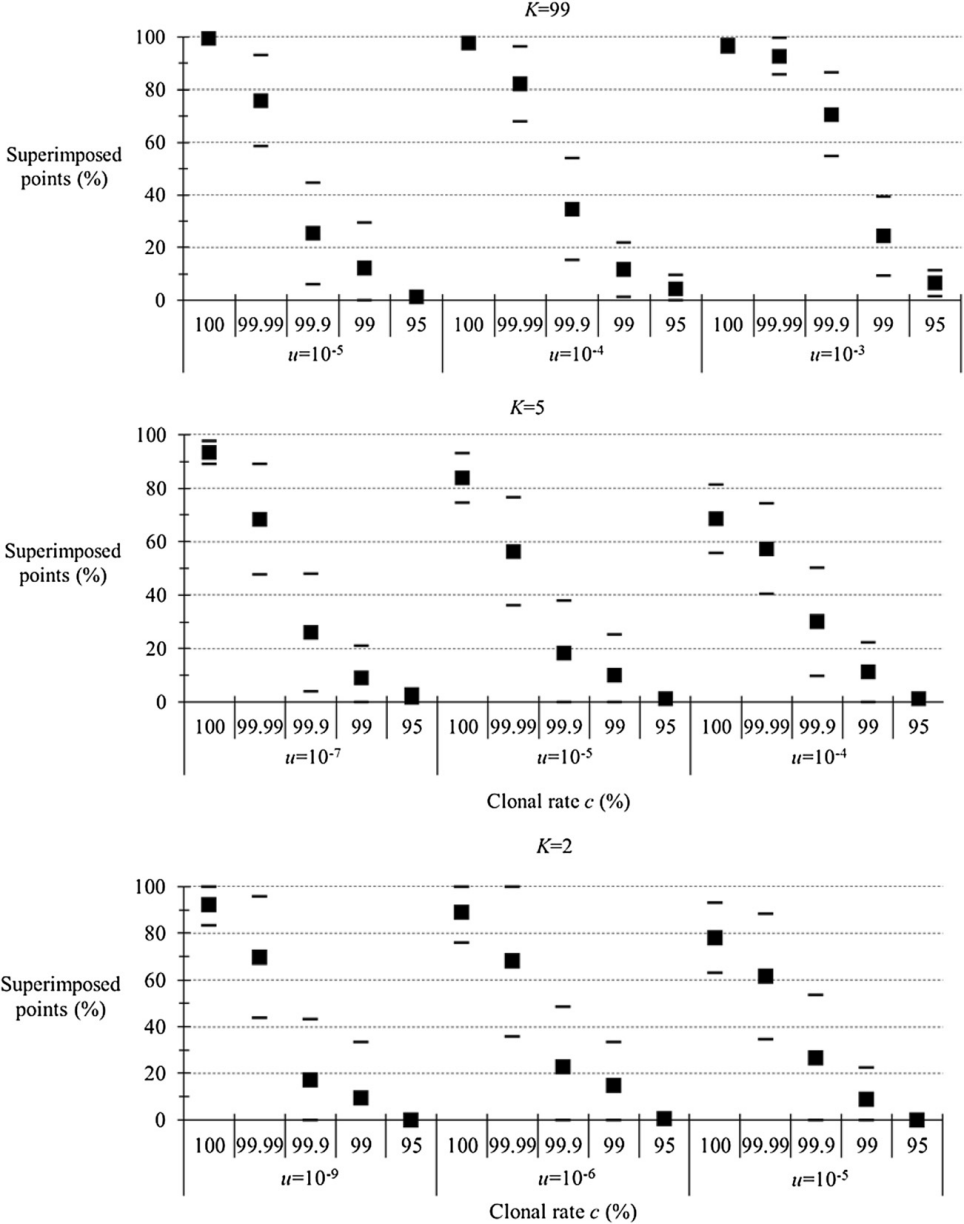
**Proportion of superimposed points (in percent) between expected and observed *F***_**IS **_**for different levels (percent) of clonality (*c*) for different mutation rates (*u*) and different degrees of homoplasy (*K*** **= 99, *K*** **= 5, *K*** **= 2) in an island model of migration.**

The results are presented in Figure 4. With little homoplasy (*K* = 99), high mutation rate (*u* = 10^−3^) has some impact. Best discrimination between rare sex and full clonality is observed for lower mutation rates (10^−4^, 10^−5^). These optimal values remain in the range of somatic (asexual) mutations observed for microsatellite loci. For an American gymnosperm tree, the estimated somatic mutation rate for microsatellites was 6.3 × 10^−4^ mutations per locus per generation, with a 95% confidence interval of 3.03 × 10^−5^ to 4.0 × 10^−3^ mutations per locus [39]. The mean rate of allele length alterations within [TC]_n_ or [AG]_n_ microsatellite loci was 6.2 × 10^−6^ mutations/cell generation in human lymphoblastoid cells [40], with a 95% confidence interval of 2.9 × 10^−6^ to 9.4 × 10^−6^. In the yeast *Aspergillus fumigatus*, average microsatellite loci mutation rate was 2.97 × 10^−4^[41], a value comparable to that obtained for *A. flavus* (2.42 × 10^−4^) [42].

For *K* = 5, optimal discrimination is observed for *u* = 10^−7^. This fits what is expected for allozyme loci. Mutation rates at allozyme loci for functional alleles are usually estimated around 10^−6^ and 10^−8^ mutations per generation [43], a third of which are seen after electrophoresis [44].

With maximum homoplasy (*K* = 2), best discrimination occurs for the lowest mutation rate (10^−9^), consistently with classical SNP mutation rates [45]. Indeed, due to low mutation rates and higher frequency of transitions as compared to transversions, SNP are generally considered as biallelic markers [45,46]. Here, clonal rates of 99.99% and 100% become difficult to distinguish from each other (as for other marker kinds).

### Discriminating rare sex from amplification problems (allelic dropouts and null alleles)

**Figure 5:**
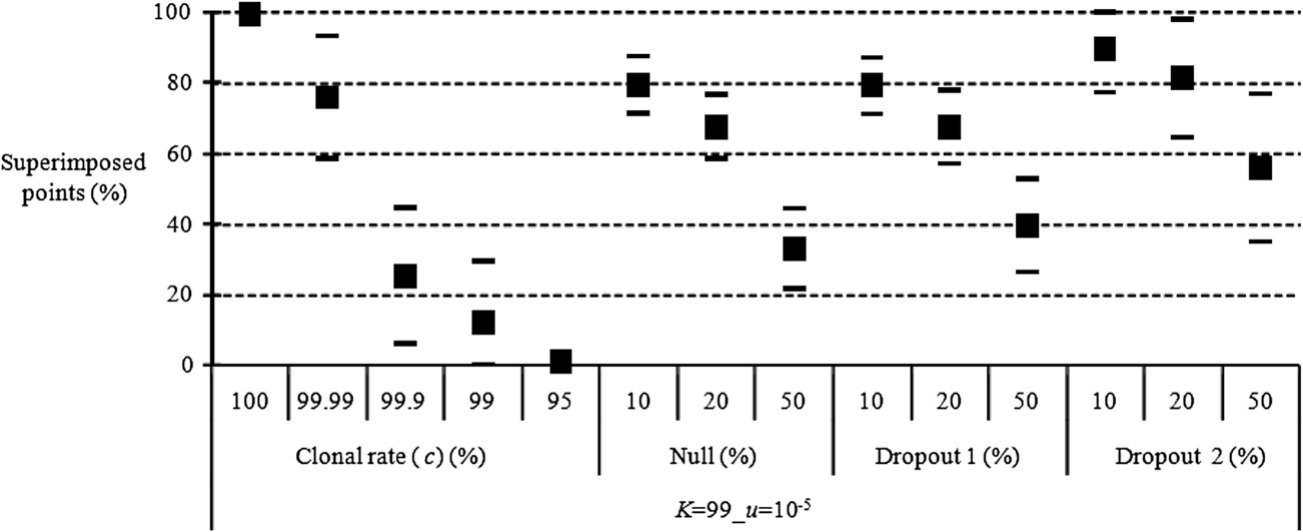
**Proportion of superimposed points (in percent) between expected and observed *F***_**IS **_**for different levels of clonality (Clonal rate, in percent), for different proportions of allelic dropouts with model 1 and model 2 (Dropout 1 and Dropout 2) and of null alleles (Null) in an island model of migration with *c*** **= 1, *K*** **= 99, *m*** **= 0.01 and *u*** **= 10**^**−5**^**.**

The results are presented in Figure 5. We note that allelic dropouts and null alleles have similar consequences regardless of dropout models. As can be seen from Figure 5, for a proportion of 10 to 20% amplification problems, the proportions of superimposed points are of the same order of magnitude as those observed with 99.99% clonality, but significantly different from those observed with *c* = 99.9%. We also observe that with 50% of amplification problems, the effects of these alleles will be very difficult to distinguish from rare events of sex, at least for *c ≥* 99%.

### Analyses of real data sets

**Figure 6:**
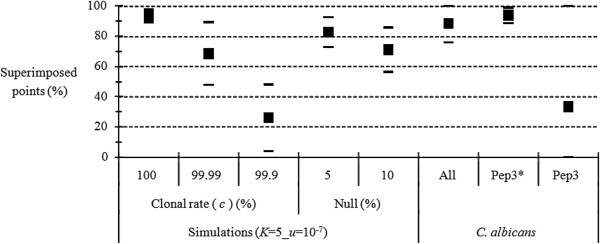
**Proportion of superimposed points (in percent) between expected and observed *F***_**IS **_**corresponding to *Candida albicans ***[27]** as compared to the proportions of superimposed points obtained by simulations with *K*** **= 5, *u*** **= 10**^**−7**^**, *m*** = 0.01, different levels (percent) of clonality (Clonal rate) and various proportions of null alleles (“Null”) in an island migration model. For the *C. albicans *data, analyses concerned all polymorphic loci (All), all polymorphic loci but locus Pep3 (Pep3*) and Pep3 taken alone (Pep3).

In an attempt to refine the *F*_IS_ estimate in *C. albicans* populations [27], seven loci (out of 14) that were suspected to display null alleles were removed from the data set. Comparing the data of *C. albicans* to simulations for which *K* = 5 and *u* = 10^−7^ (see above), our results show that these data are consistent with those of strictly clonal organisms (Figure 6). Loci suspected of presenting null alleles only weakly alter the signal. In fact, removal of a single locus from the data set (Pep3) is enough to perfectly fit theoretical expectations under full clonality. This confirms the need to exclude this locus for *F*_IS_ estimation before proceeding to demographic inferences, but invalidates the exclusion of the six other incriminated loci [27], whose unique flaw was their weak polymorphism.

For trypanosome data, resulting from microsatellite markers, we chose to compare the data with simulations with *K* = 99 and *u* = 10^−5^.

**Figure 7:**
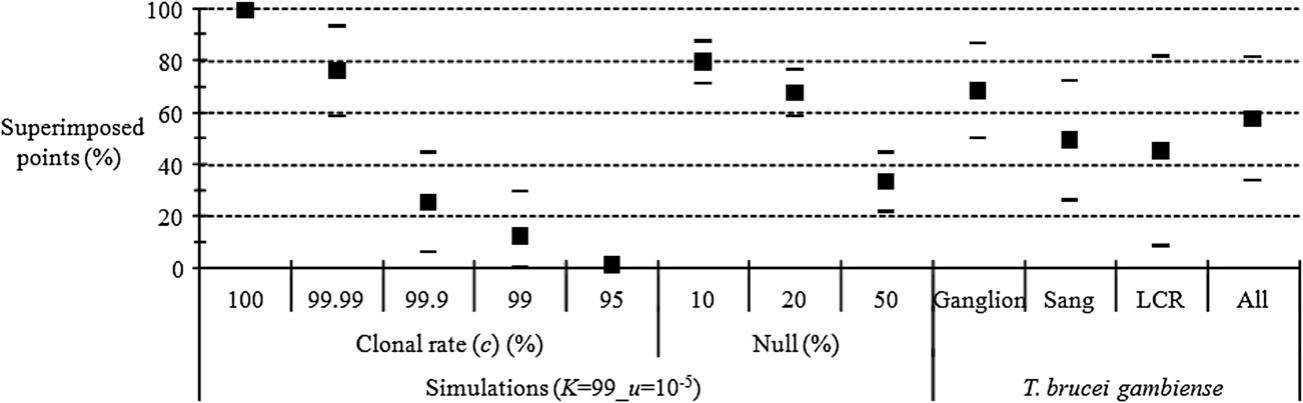
**Proportion of superimposed points (in percent) between expected and observed *F***_**IS **_**corresponding to *Trypanosoma brucei gambiense ***[28]** compared to the proportion of superimposed points obtained by simulations with *K*** **= 99, *u*** **= 10**^**−5**^**, *m*** **= 0.01, different levels of clonality (Clonal rate) and various proportions of null alleles (Nuls in%) in an island model of migration. ***T. brucei gambiense* DNA was amplified from different fluids: lymph of cervical node (Lymph), blood (Blood) and Cerebrospinal Fluid (CSF).

For *T. brucei gambiense*[28], the results are broadly consistent with very rare events of sex (one recombined zygote out of 10000) or amplification problems (e.g. null alleles) varying from 10 to 20% for lymph, less than 50% for blood and about 50% for cerebrospinal fluid (CSF) (Figure 7).

If we set *P*_*n*_ as the proportion of null alleles in a data set, *N*_*b*_ as the number of blank genotypes and *N* as the total number of genotypes (sample size multiplied by the number of loci), then we should have, in a clonal population with weak homoplasy:

(4) $$\begin{array}{l} P_{n}\approx \frac{2N_{b}+p_{n}\left(N-N_{b}\right)}{2N}\\ 2NP_{n}=2N_{b}+p_{n}\left(N-N_{b}\right)\\ 2NP_{n}-p_{n}\left(N-N_{b}\right)=2N_{b}\\ P_{n}\left[2N-\left(N-N_{b}\right)\right]=2N_{b}\\ P_{n}=\frac{2N_{b}}{N+N_{b}} \end{array}$$

Knowing that *N* = 582 for lymph and blood and *N* = 180 for CSF, that *N*_*b*_ = 26, 160 and 103 for lymph, blood and CSF, respectively, equation 4 thus allows obtaining a proxy for the proportion of null alleles in the data sets; here about 8.5%, 42.8% and 72.6%, respectively for the different fluids (lymph, blood and CSF), assuming all blanks are indeed homozygous nulls.

In pure clonal populations with null alleles and low homoplasy, the number of individuals seen homozygous (N*) is:

(5) $$N^{*}\approx P_{n}\left(N-N_{b}\right)$$

In *T. brucei gambiense*, the number of observed homozygotes was 39, 85 and 26 for lymph, blood and CSF respectively, while the expected homozygotes (N*) were 45.5, 178.4 and 55.3 respectively. The *P*-values resulting from the comparison made by the exact unilateral binomial test (the number of homozygous profiles observed does not exceed the expected number calculated with the observed number of blanks) between expected and observed data were 0.8348, 1 and 1 for the lymph, blood and CSF respectively. In fact, there are significantly less observed homozygotes than expected, which tends to suggest that many blanks are due to total amplification failure (not enough DNA), rather than to true null alleles. If we refer to Figure 7, we then cannot exclude very rare events of sex to explain *T. brucei gambiense* data. However, the means are consistent with significant proportions (10-40%) of amplification problems in a completely clonal population. The excessive number of observed blanks provides an additional argument in favor of this interpretation. This would make this data set the result from a combined effect of nulls and of our Dropout 2 model.

**Figure 8:**
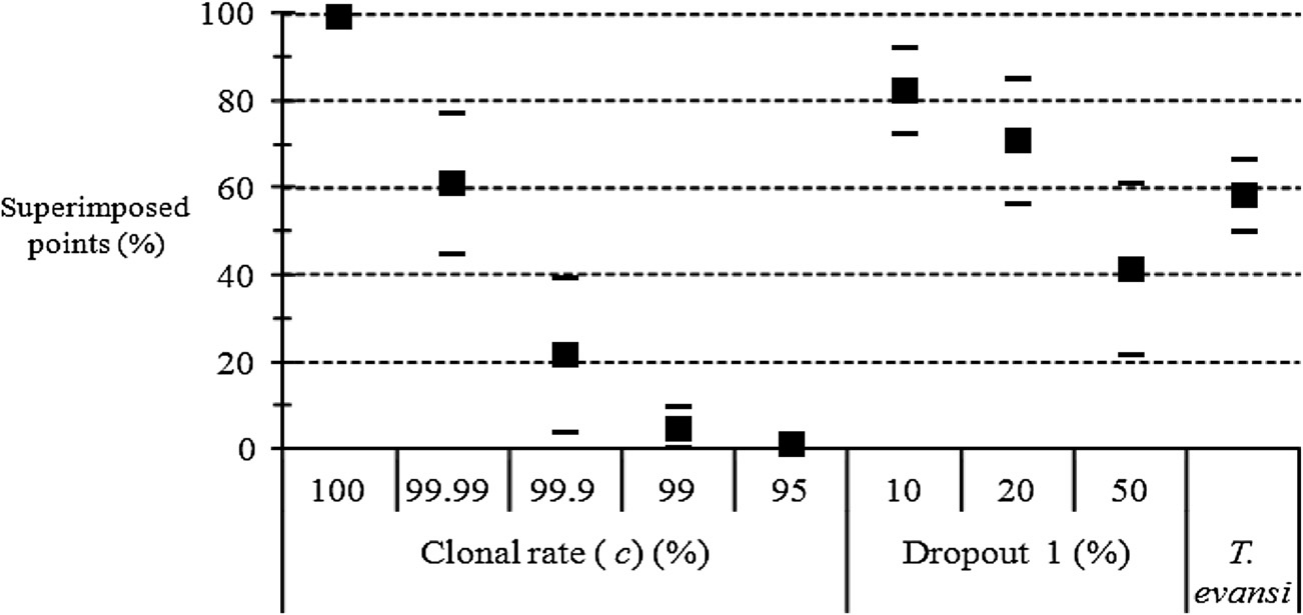
**Proportion of superimposed points (in percent) between expected and observed *F***_**IS **_**corresponding to *Trypanosoma evansi ***[32]** compared to the proportions of superimposed points (in percent) obtained by simulations of a two-dimension stepping-stone model with *K*** **= 99, *u*** **= 10**^**−5**^**, *m*** **= 0.05, various clonal rates (Clonal rate) and proportions of allelic dropouts (model 1) (Dropout 1).**

The genotypic data obtained for *T. evansi* did not contain any missing data [32]. Therefore, neither null alleles nor Dropout 2 model can in principle be incriminated to explain the substantial number of homozygotes observed. By examining Figure 8, we see that these data are consistent with more than 20% of allelic dropouts or with *c* = 99.99%.

**Figure 9:**
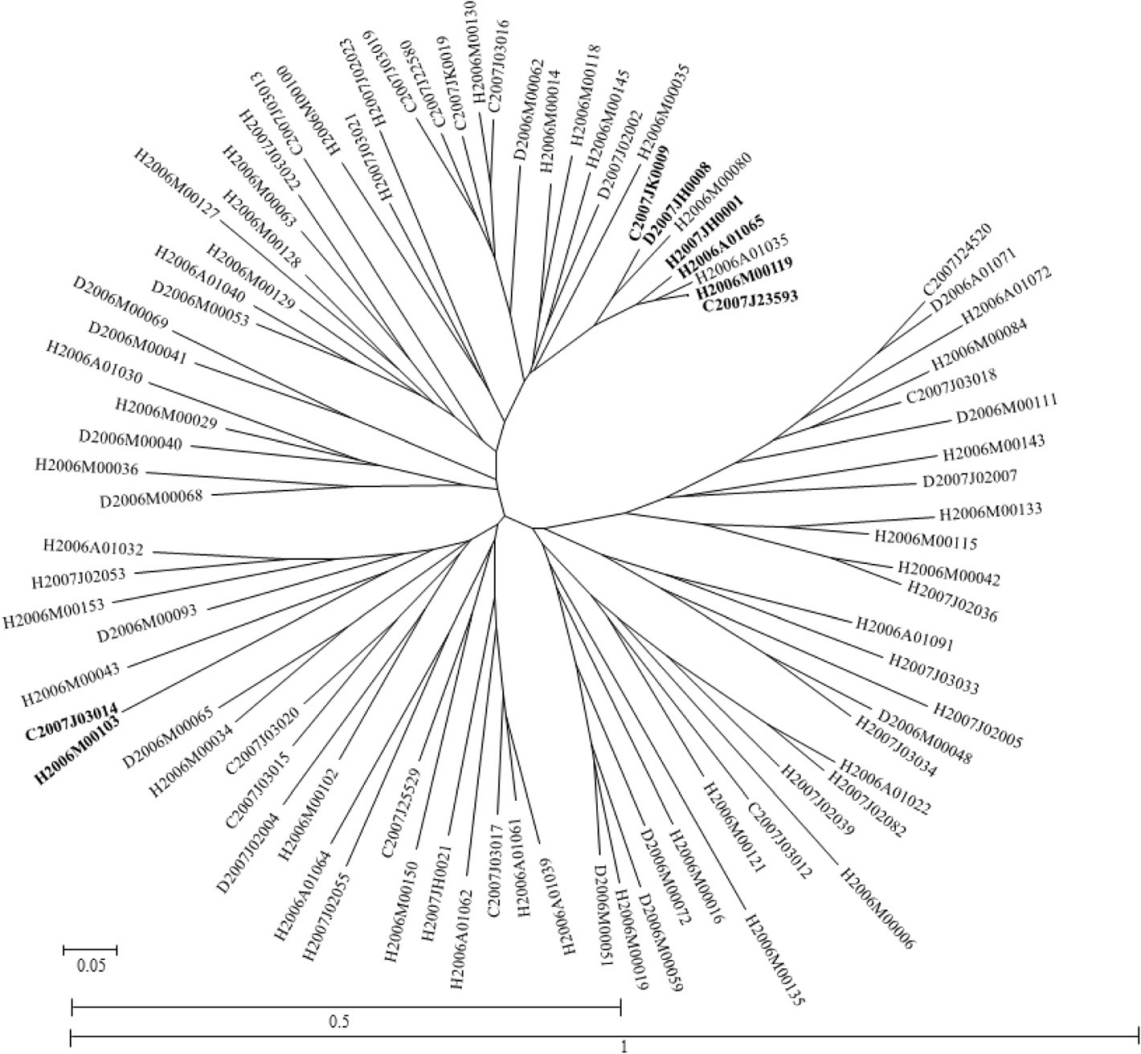
**Dendrogram based on Cavalli-Sforza and Edwards chord distance **[36]** between microsatellite profiles obtained from *Trypanosoma congolense *samples **[33]**.** The first letter represents the host species (C for cow, H for horse and D for donkey), followed by the year and the number of individuals. Identical genotypes are in bold type.

No superimposing was observed with *T. congolense* data (results not presented). There are a total of 115 missing data in this sample of 756 genotypes. Applying equation (3) to these data, we obtained 23.33% of expected null alleles. This amounts to 150 expected homozygous individuals against 367 observed in the data. The *P*-values resulting from the comparison made by the exact unilateral binomial test (the number of homozygous profiles observed does not exceed the expected number calculated with the observed number of blanks) between the number of observed and expected homozygous profiles was highly significant (*P*-value < 10^−4^). So, there are more observed homozygous profiles in the data sets than expected. Null alleles therefore cannot explain the observed proportion of homozygotes (49%). Even if we imagine a mixed system of dropouts and nulls, the proportion of alleles with a problem of amplification that might explain the observed homozygosity would be about 64%. Yet we know that at this percentage, the average proportion of superimposed points obtained in our simulations (not shown) is not zero as it is here. These results would thus suggest frequent and inbred sex (selfing) for this trypanosome species, as concluded by the authors [33]. Nevertheless, the very high variance of *F*_IS_ from one locus to the other does not support this hypothesis. Moreover, if we refer to the dendrogram in Figure 9, the genetic distances between many pairs of individuals are unexpectedly high with a mean = 0.634 ± 0.03. This is quite unexpected from individuals of the same species sampled in the same site and genotyped at seven microsatellite loci. Amplification hazards and perhaps unresolved species coexistence probably led to this inconsistent and therefore impossible to interpret data set.

**Figure 10:**
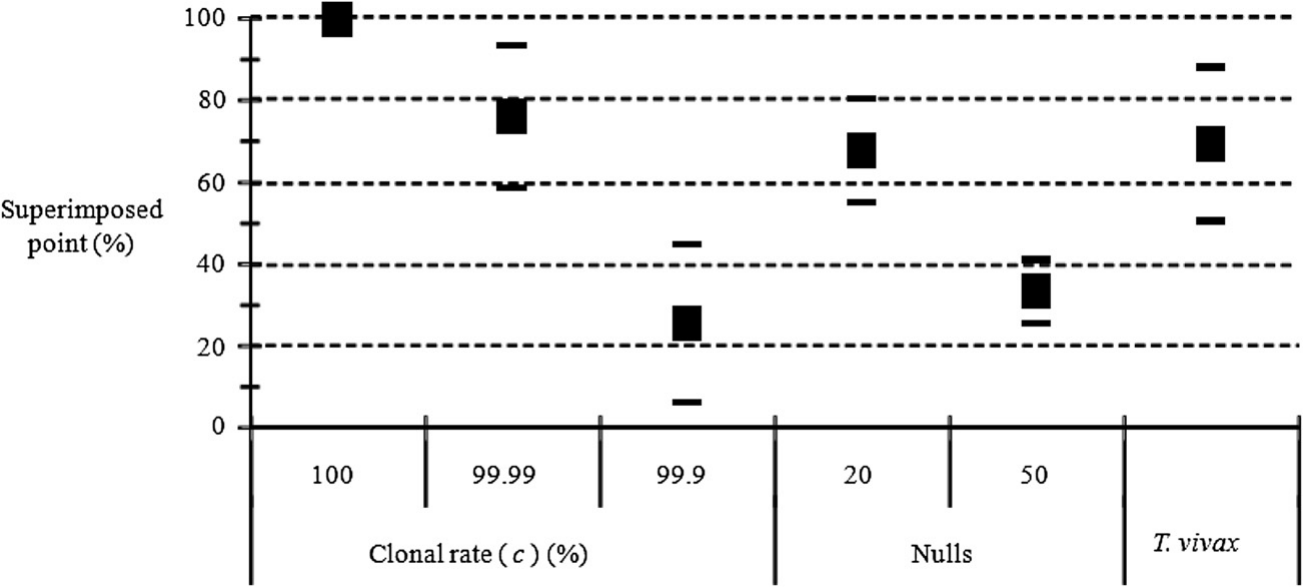
**Proportion of superimposed points (in percent) between expected and observed *F***_**IS **_**corresponding to *Trypanosoma vivax ***[34]**. Results are compared to the proportions of superimposed points obtained by simulations with *K*** **= 99, *m*** **= 0.01 and *u*** **= 10**^**−5 **^**in an island model.** The simulations concerned different levels (percent) of clonality (“Clonal rate”) and various proportions of null alleles (Null) in the data from entirely clonal populations.

The proportion of superimposed points obtained with *T. vivax*[34], is consistent with those of clonal populations with 20% of amplification problems or very rare sex (*c* = 99.99%) (Figure 10).

## Discussion

The first result is that low migration rates lower the discriminating power of our criterion, but only for extremely rare events of sexual recombination (1 per 10000). Some difficulties arise when the mutation rate increases, so that discrimination between very rare events of sex (one out of 10000 reproduction events) and pure clonality becomes problematic. Given the likely size of populations of the organisms under study, in particular trypanosomes, and given sample sizes usually available, the detection of 1 recombination event over 10000 reproductive events appears insignificant. When the lower mutation rates documented for microsatellite in clones are used [39,41,42], the discriminating power remains very good. We have also seen that markers with maximum homoplasy (*K* = 2) and high mutation rate (*u* = 10^−5^) can present difficulties, which might exclude SNPs that are functionally bi-allelic [45]. Given that SNPs mutation rates are around 10^−9^[45], such difficulties will not hold in most situations (though highly variable markers perform better for many other reasons). When *K* = 5, which may correspond to allozymes, the difficulties only appear for mutation rates (u ≥ 10^−4^) that will hardly be met for such markers, for which *u* = 10^−7^ appears more likely [43,44].

A most serious problem arises after a given threshold of amplification difficulties (50%), where discriminating between amplification problems and sexual events (i.e. 1 to 5%) becomes difficult.

We have confirmed total clonality with some null alleles at a single locus for *C. albicans*. For the other six suspected loci [27], the difficulties probably came from the combined effects of substantial homoplasy and weak polymorphism at these loci. Estimating *F*_IS_ with the 13 remaining loci thus provides the best tool for further inferences.

We have confirmed total clonality with a significant proportion of null alleles and/or allelic dropouts for Guinean *T. brucei gambiense* from body fluids, with more problems in the CSF than in the blood, and most success for lymph amplified samples. These observations are in line with the discussion found in the initial paper [47]. The advice here would have been to repeat DNA amplifications for those loci and samples that appeared homozygous or blank. This was indeed done and revealed that most of those genotypes were in fact true heterozygotes [48].

For African trypanosomes, recombination (if any) occurs in the salivary glands of tsetse flies and *T. evansi* has lost the ability to be cyclically transmitted by tsetse flies [30], that are absent anyway from the investigated zone presented here [32]. Combined with the absence of missing data, our criterion argues for allelic dropouts (model 1) up to 20-50% in this species. This is consistent with a recent study [31], where isolated *T. evansi* were genotyped using different loci from those presented here, showing perfect adequacy with a purely clonal population with 100% of superimposed points (not shown). Here the advice would be using such loci to genotype Sudanese isolates again.

*T. congolense* does not stay in the salivary glands of the tsetse fly [49] where sexual recombination events take place [30,50,51]. One would thus expect a clonal reproduction for this trypanosome species as already advocated [52]. However, we found a complete absence of superimposed points between expected and observed *F*_IS_ in this study. Missing data and suspected null alleles cannot explain this situation. This lack of superimposed points might therefore be the signature of an important part played by sexual recombination as already invoked in the original article [33]. However, the high number of amplification failures encountered in this study, combined with the large variance of *F*_IS_ across loci and extraordinary genetic distances between most isolates, suggest the need for a better control of the molecular and/or ecological events that led to these surprising observations. Within the same sexually recombining species, within the same geographical site and for microsatellite loci, which are known for their homoplasy (even if moderate), observing such divergences between individuals is unexpected, not to say inconsistent. However, these results could be explained by aneuploidy, in which case each chromosome passes frequently through a haploid state, which purges heterozygosity and leads to a heterozygous deficiency. This hypothesis still remains to be verified for *T. congolense*, since many recent studies have demonstrated a diploid state in African trypanosomes [53].

The case of *T. vivax* is typical of variance problems met with small sample sizes (only 31 available genotypes). Here, given the negative value of all *F*_IS_ (unexpected if there was any sex), amplification problems (null alleles) are probably the cause of the observed variance across loci. Because here most loci are affected, primers probably need to be redesigned or new loci tested before getting access to accurate estimates of *F*_IS_ and hence before being able to use it for inferences.

Allelic dropouts and null alleles in clonal organisms, may display the same consequences as those of extremely rare sex (less than 5%). In this study, the method based on the relationship between *H*_*S*_ and *F*_IS_ under the assumption of clonal reproduction has proved a useful criterion for deciding if an unusual homozygosity could be resulting from technical problems (allelic dropouts and/or null alleles) in clonal organisms, provided that the frequency of the latter does not exceed 50%. Our criterion easily discriminates between rare sex (at least above 1/10000) and hidden alleles. As discussed above, a 1/10000 sexual recombination event will rarely be accessible in most situations and our criterion is just a tool indicating if supplementary genotyping is required, in particular for homozygous and missing phenotypes. The presence of blank genotypes can represent strong support in that respect but will only be useful in null allele cases and Dropout 2 kind of models. Allelic dropouts are indeed unlikely to generate many homozygous profiles if any [19-21]. It is worth noting that this tool does not provide the proportion of hidden alleles in the real datasets of clones, which is another interesting, though much more complex issue. We have proposed a rough solution in case of null alleles using the proportion of missing data, assuming all are null homozygotes. Nevertheless, the technique presented here does not represent a palliative but a useful decision criterion that can lead to the elimination of problematic loci, the re-amplification of homozygous and/or missing genotypes, or to the design of new sets of primers.

## Conclusion

Our criterion of superimposing between the *F*_IS_ expected under clonality and the observed *F*_IS_ has indeed been effective when amplification difficulties occur in low to moderate frequencies (20-30%), because the relationship between *F*_IS_ and *H*_S_ disappears significantly more rapidly with sexual recombination than with the presence of hidden alleles. Generally, when the criterion is compatible with 99.99% of sex or hidden alleles (between 60% and 100% of superimposed points) it could be worth rejecting those loci responsible for the high variance (when it is possible), or repeating DNA amplifications on those extracts that gave homozygous profiles and/or missing data, or redesigning other primer pairs and/or look for other loci.

## Abbreviations

DNA: Deoxyribose nucleic acid; CSF: Cerebrospinal fluid; IAM: Infinite allele model; KAM: K allele model; PCR: Polymerase chain reaction; SMM: Strict stepwise mutation model; SNP: Single Nucleotide polymorphism.

## Competing interests

We do not have any financial or non-financial competing interests concerning this manuscript.

## Authors’ contributions

MS has contributed to simulated data acquisition, analyses and interpretation and wrote the paper. JK has contributed to the conception of this study and has been involved in manuscript writing. VJ has contributed to the conception of this study and has been involved in manuscript writing. AMGB has contributed to the conception of this study and has been involved in manuscript writing. FJA has contributed to the conception of this study and has been involved in manuscript writing. TDM has supervised the whole work, has contributed to the conception of this study, to analysis and interpretation of data and he has been involved in manuscript writing. All authors read and approved the final version of the manuscript.

## Supplementary Material

Additional file 1: Figure S1 Proportion of superimposed points (in percent) between expected and observed *F*_IS_ for different levels (percent) of clonality (*c*) and different percentages of null alleles (Null): The results where all loci and subsamples were kept (even those with *H*_S_ <05) and the same after excluding loci displaying *H*_S_<0.5 are shown to demonstrate the benefit of excluding such data. The proportions of superimposed points have been obtained by simulations with *K*=5, *m*=0.01 and *u*=10^−5^ in an island model. Click here for file

## References

[B1] De Meeûs T McCoy KD Prugnolle F Chevillon C Durand P Hurtrez-Boussès S Renaud F Population genetics and molecular epidemiology or how to “débusquer la bête” Infect Genet Evol 2007 7 308 332 16949350 10.1016/j.meegid.2006.07.003

[B2] Prugnolle F De Meeûs T The impact of clonality on parasite population genetic structure Parasite 2008 15 455 457 18814722 10.1051/parasite/2008153p455

[B3] McCoy KD The population genetic structure of vectors and our understanding of disease epidemiology Parasite 2008 15 444 448 18814720 10.1051/parasite/2008153444

[B4] Criscione CD Poulin R Blouin MS Molecular ecology of parasites: elucidating ecological and microevolutionary processes Mol Ecol 2005 14 2247 2257 15969711 10.1111/j.1365-294X.2005.02587.x

[B5] De Meeûs T McCoy KD Guégan JF, Choisy M La génétique des populations comme outil en épidémiologie Introduction à l’Epidémiologie Intégrative des Maladies Infectieuses et Parasitaires 2009 Bruxelles: De Boek Université 277 310

[B6] De Meeûs T Renaud F Parasites within the new phylogeny of eukaryotes Trends Parasitol 2002 18 247 251 12036736 10.1016/s1471-4922(02)02269-9

[B7] Wright S The interpretation of population structure by F-statistics with special regard to system of mating Evolution 1965 19 395 420

[B8] Balloux F Lehmann L De Meeûs T The population genetics of clonal and partially clonal diploids Genetics 2003 164 1635 1644 12930767 10.1093/genetics/164.4.1635 PMC1462666

[B9] De Meeûs T Lehmann L Balloux F Molecular epidemiology of clonal diploids: A quick overview and a short DIY (do it yourself) notice Infect Genet Evol 2006 6 163 170 16290062 10.1016/j.meegid.2005.02.004

[B10] Cockerham CC Variance of gene frequencies Evolution 1969 23 72 84 10.1111/j.1558-5646.1969.tb03496.x 28562963

[B11] Cockerham CC Analysis of gene frequencies Genetics 1973 74 679 700 17248636 10.1093/genetics/74.4.679 PMC1212983

[B12] Balloux F EASYPOP (version 2.01): A computer program for population genetics simulations J Hered 2001 92 301 302 11447253 10.1093/jhered/92.3.301

[B13] Rousset F Equilibrium values of measures of population subdivision for stepwise mutation processes Genetics 1996 142 1357 1362 8846911 10.1093/genetics/142.4.1357 PMC1207131

[B14] De Meeûs T Initiation à la génétique des populations naturelles: Applications aux parasites et à leurs vecteurs 2012 Marseille: IRD Editions

[B15] Wright S The genetical structure of populations Ann Eugen 1951 15 323 354 24540312 10.1111/j.1469-1809.1949.tb02451.x

[B16] Kimura M Weiss GH The Stepping Stone Model of Population Structure and the Decrease of Genetic Correlation with Distance Genetics 1964 49 561 576 17248204 10.1093/genetics/49.4.561 PMC1210594

[B17] Rousset F Genetic differentiation and estimation of gene flow from F-statistics under isolation by distance Genetics 1997 145 1219 1228 9093870 10.1093/genetics/145.4.1219 PMC1207888

[B18] Wang C Schroeder KB Rosenberg NA A maximum-likelihood method to correct for allelic dropout in microsatellite data with no replicate genotypes Genetics 2012 192 651 669 22851645 10.1534/genetics.112.139519 PMC3660999

[B19] Johnson PC Haydon DT Maximum-likelihood estimation of allelic dropout and false allele error rates from microsatellite genotypes in the absence of reference data Genetics 2007 175 827 842 17179070 10.1534/genetics.106.064618 PMC1800638

[B20] Wang J Sibship reconstruction from genetic data with typing errors Genetics 2004 166 1963 1979 15126412 10.1534/genetics.166.4.1963 PMC1470831

[B21] Miller CR Joyce P Waits LP Assessing allelic dropout and genotype reliability using maximum likelihood Genetics 2002 160 357 366 11805071 10.1093/genetics/160.1.357 PMC1461941

[B22] Corley LS Blankenship JR Moore AJ Genetic variation and asexual reproduction in the facultatively parthenogenetic cockroach Nauphoeta cinerea: implications for the evolution of sex J Evolution Biol 2001 14 68 74 10.1046/j.1420-9101.2001.00254.x 29280573

[B23] Weir BS Cockerham CC Estimating F-statistics for the analysis of population structure Evolution 1984 38 1358 1370 10.1111/j.1558-5646.1984.tb05657.x 28563791

[B24] Nei M Chesser RK Estimation of fixation indices and gene diversities Ann Hum Genet 1983 47 253 259 6614868 10.1111/j.1469-1809.1983.tb00993.x

[B25] Goudet J Fstat (ver. 2.9.4), a program to estimate and test population genetics parameters 2003 Available from http://www2.unil.ch/popgen/softwares/fstat.htm Updated from Goudet (1995)

[B26] Goudet J FSTAT (Version 1.2): A computer program to calculate F-statistics J Hered 1995 86 485 486

[B27] Nébavi F Ayala FJ Renaud F Bertout S Eholié S Moussa K Mallié M De Meeûs T Clonal population structure and genetic diversity of *Candida albicans* in AIDS patients from Abidjan (Côte d’Ivoire) Proc Natl Acad Sci U S A 2006 103 3663 3668 16501044 10.1073/pnas.0511328103 PMC1450139

[B28] Kaboré J Macleod A Jamonneau V Ilboudo H Duffy C Camara M Camara O Belem AM Bucheton B De Meeus T Population genetic structure of Guinea *Trypanosoma brucei gambiense* isolates according to host factors Infect Genet Evol 2011 11 1129 1135 21515408 10.1016/j.meegid.2011.04.011

[B29] Koffi M De Meeûs T Bucheton B Solano P Camara M Kaba D Cuny G Ayala FJ Jamonneau V Population genetics of *Trypanosoma brucei gambiense*, the agent of sleeping sickness in Western Africa Proc Natl Acad Sci U S A 2009 106 209 214 19106297 10.1073/pnas.0811080106 PMC2629214

[B30] Gibson W Resolution of the species problem in African trypanosomes Int J Parasitol 2007 37 829 838 17451719 10.1016/j.ijpara.2007.03.002

[B31] McInnes LM Dargantes AP Ryan UM Reid SA Microsatellite typing and population structuring of Trypanosoma evansi in Mindanao, Philippines Vet Parasitol 2012 187 129 139 22230026 10.1016/j.vetpar.2011.12.010

[B32] Salim B De Meeûs T Bakheit MA Kamau J Nakamura I Sugimoto C Population genetics of *Trypanosoma evansi* from Camel in Sudan PLoS Negl Trop Dis 2011 5 e1196 21666799 10.1371/journal.pntd.0001196 PMC3110163

[B33] Morrison LJ Tweedie A Black A Pinchbeck GL Christley RM Schoenefeld A Hertz-Fowler C MacLeod A Turner CM Tait A Discovery of mating in the major African livestock pathogen *Trypanosoma congolense* PLoS One 2009 4 e5564 19440370 10.1371/journal.pone.0005564 PMC2679202

[B34] Duffy CW Morrison LJ Black A Pinchbeck GL Christley RM Schoenefeld A Tait A Turner CM MacLeod A *Trypanosoma vivax* displays a clonal population structure Int J Parasitol 2009 39 1475 1483 19520081 10.1016/j.ijpara.2009.05.012

[B35] R-Development-core-team R: A Language and Environment for Statistical Computing R Foundation for Statistical Computing, Vienna, Austria http://www.R-project.org, ISBN 3-900051-07-0 2010

[B36] Cavalli-Sforza LL Edwards AWF Phylogenetic analysis: model and estimation procedures Am J Hum Genet 1967 19 233 257 6026583 PMC1706274

[B37] Dieringer D Schlötterer C Microsatellite analyser (MSA): a platform independent analysis tool for large microsatellite data sets Mol Ecol Notes 2002 3 167 169

[B38] Tamura K Peterson D Peterson N Stecher G Nei M Kumar S MEGA5: molecular evolutionary genetics analysis using maximum likelihood, evolutionary distance, and maximum parsimony methods Mol Biol Evol 2011 28 2731 2739 21546353 10.1093/molbev/msr121 PMC3203626

[B39] O'Connell LM Ritland K Somatic mutations at microsatellite loci in western Redcedar (Thuja plicata: Cupressaceae) J Hered 2004 95 172 176 15073234 10.1093/jhered/esh024

[B40] Hile SE Yan G Eckert KA Somatic mutation rates and specificities at TC/AG and GT/CA microsatellite sequences in nontumorigenic human lymphoblastoid cells Cancer Res 2000 60 1698 1703 10749142

[B41] Klaassen CH Gibbons JG Fedorova ND Meis JF Rokas A Evidence for genetic differentiation and variable recombination rates among Dutch populations of the opportunistic human pathogen *Aspergillus fumigatus* Mol Ecol 2012 21 57 70 22106836 10.1111/j.1365-294X.2011.05364.x PMC3258581

[B42] Grubisha LC Cotty PJ Genetic isolation among sympatric vegetative compatibility groups of the aflatoxin-producing fungus *Aspergillus flavus* Mol Ecol 2010 19 269 280 20025654 10.1111/j.1365-294X.2009.04467.x

[B43] Hardy OJ de Loose M Vekemans X Meerts P Allozyme segregation and inter-cytotype reproductive barriers in the polyploid complex Centaurea jacea Heredity (Edinb) 2001 87 136 145 11703503 10.1046/j.1365-2540.2001.00862.x

[B44] Shaw CR How many genes evolve? Biochem Genet 1970 4 275 283 5463816 10.1007/BF00485778

[B45] Vignal A Milan D SanCristobal M Eggen A A review on SNP and other types of molecular markers and their use in animal genetics Genet Sel Evol 2002 34 275 305 12081799 10.1186/1297-9686-34-3-275 PMC2705447

[B46] Rosenberg NA Huang L Jewett EM Szpiech ZA Jankovic I Boehnke M Genome-wide association studies in diverse populations Nat Rev Genet 2010 11 356 366 20395969 10.1038/nrg2760 PMC3079573

[B47] Kaboré J Koffi M Bucheton B MacLeod A Duffy C Ilboudo H Camara M De Meeus T Belem AM Jamonneau V First evidence that parasite infecting apparent aparasitemic serological suspects in human African trypanosomiasis are *Trypanosoma brucei gambiense* and are similar to those found in patients Infect Genet Evol 2011 11 1250 1255 21530681 10.1016/j.meegid.2011.04.014

[B48] Kabore J De Meeus T Macleod A Ilboudo H Capewell P Camara M Gaston Belem AM Bucheton B Jamonneau V A protocol to improve genotyping of problematic microsatellite loci of Trypanosoma brucei gambiense from body fluids Infect Genet Evol 2013 20C 171 176 23954418 10.1016/j.meegid.2013.08.006

[B49] Hoare CB The trypanosomes of mammals. A zoological monograph 1972 Oxford: Blackwell

[B50] Gibson WC The significance of genetic exchange in trypanosomes Parasitol Today 1995 11 465 468

[B51] Tait A MacLeod A Tweedie A Masiga D Turner CMR Genetic exchange in *Trypanosoma brucei*: Evidence for mating prior to metacyclic stage development Mol Biochem Parasit 2007 151 133 136 10.1016/j.molbiopara.2006.10.009 PMC2311417 17134768

[B52] Tibayrenc M Kjellberg F Arnaud J Oury B Brenière SF Dardé ML Ayala FJ Are eukaryotic microorganisms clonal or sexual? A population genetics vantage Proc Natl Acad Sci U S A 1991 88 5129 5133 1675793 10.1073/pnas.88.12.5129 PMC51825

[B53] MacLeod A Tweedie A McLellan S Taylor S Hall N Berriman M El-Sayed NM Hope M Turner CM Tait A The genetic map and comparative analysis with the physical map of Trypanosoma brucei Nucleic Acids Res 2005 33 6688 6693 16314301 10.1093/nar/gki980 PMC1297707

