# Supplementary figures and images for: Null allele, allelic dropouts or rare sex detection in clonal organisms: simulations and application to real data sets of pathogenic microbes
Source: Parasit Vectors. 2014 Jul 15;7:331. doi: 10.1186/1756-3305-7-331 (PMC4223633; doi:10.1186/1756-3305-7-331)

Figure S1


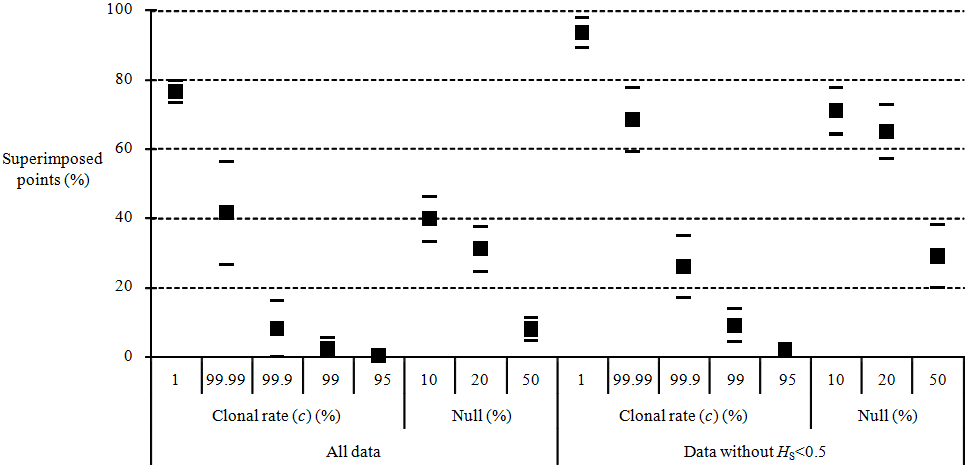

Supplement: Additional file 1: Figure S1 — Proportion of superimposed points (in percent) between expected and observed FIS for different levels (percent) of clonality (c) and different percentages of null alleles (Null): The results where all loci and subsamples were kept (even those with HS <05) and the same after excluding loci displaying HS<0.5 are shown to demonstrate the benefit of excluding such data. The proportions of superimposed points have been obtained by simulations with K=5, m=0.01 and u=10−5 in an island model. [file 1756-3305-7-331-S1.docx]
